# Unveiling the Emergent Traits of Chiral Spin Textures in Magnetic Multilayers

**DOI:** 10.1002/advs.202103978

**Published:** 2022-01-02

**Authors:** Xiaoye Chen, Ming Lin, Jian Feng Kong, Hui Ru Tan, Anthony K.C. Tan, Soong‐Geun Je, Hang Khume Tan, Khoong Hong Khoo, Mi‐Young Im, Anjan Soumyanarayanan

**Affiliations:** ^1^ Institute of Materials Research & Engineering Agency for Science Technology & Research (A*STAR) Singapore 138634 Singapore; ^2^ Data Storage Institute Agency for Science Technology & Research (A*STAR) Singapore 138634 Singapore; ^3^ Institute of High Performance Computing Agency for Science Technology & Research (A*STAR) Singapore 138632 Singapore; ^4^ Center for X‐Ray Optics Lawrence Berkeley National Laboratory Berkeley CA 94720 USA; ^5^ Department of Physics National University of Singapore Singapore 117551 Singapore

**Keywords:** electron microscopy, magnetic films, micromagnetics, skyrmions, X‐ray microscopy

## Abstract

Magnetic skyrmions are topologically wound nanoscale textures of spins whose ambient stability and electrical manipulation in multilayer films have led to an explosion of research activities. While past efforts focused predominantly on isolated skyrmions, recently ensembles of chiral spin textures, consisting of skyrmions and magnetic stripes, are shown to possess rich interactions with potential for device applications. However, several fundamental aspects of chiral spin texture phenomenology remain to be elucidated, including their domain wall (DW) structure, thermodynamic stability, and morphological transitions. Here the evolution of these textural characteristics are unveiled on a tunable multilayer platform—wherein chiral interactions governing spin texture energetics can be widely varied—using a combination of full‐field electron and soft X‐ray microscopies with numerical simulations. With increasing chiral interactions, the emergence of Néel helicity, followed by a marked reduction in domain compressibility, and finally a transformation in the skyrmion formation mechanism are demonstrated. Together with an analytical model, these experiments establish a comprehensive microscopic framework for investigating and tailoring chiral spin texture character in multilayer films.

## Graphical abstract

Magnetic skyrmions are chiral spin textures with nanoscale size and ambient stability—promising for unconventional computing applications. Here, with increasing interfacial chiral interactions, sequential transitions in the chiral microstructure, thermodynamic stability, field evolution, and formation mechanism of skyrmions are unveiled. Using complementary microscopy techniques and an analytical model, a framework for tailoring spin texture character is established in multilayer films.

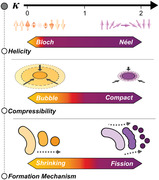

## 1 Introduction

Seminal advances in tailoring interfacial interactions in magnetic thin films have led to the room temperature (RT) stabilization of nanoscale spin textures—most notably magnetic skyrmions.^[^ ^1^, ^2^, ^3^, ^4^ ^]^ In light of past efforts on magnetic bubbles and domain walls (DWs),^[^ ^5^, ^6^ ^]^ the excitement around magnetic skyrmions stems largely from their nontrivial topology, small size, and their coupling to electrical stimuli. First, skyrmions possess a non‐zero topological charge, which emerges from the material‐specific handedness and manifests as the chirality of spins winding around their center.^[^ ^7^ ^]^ The unique spin structure of skyrmions facilitates their electrical detection,^[^ ^7^, ^8^ ^]^ while also enabling them to remain stable at sizes down to 2 nm.^[^ ^9^, ^10^ ^]^ Importantly, they can be electrically generated,^[^ ^11^, ^12^, ^13^, ^14^ ^]^ and driven at relatively high efficiency using electrical currents.^[^ ^2^, ^15^ ^]^

While sparse, isolated skyrmions in chiral multilayers are attractive for spintronic device applications, equally ripe for exploitation are denser ensembles of chiral spin textures, consisting of magnetic stripes and skyrmions. Several recent proposals look to harness these so‐called “skyrmion fabrics”^[^ ^16^, ^17^ ^]^ for reservoir computing. However, a comprehensive microscopic picture of chiral spin texture phenomenology, and their response to external stimuli, remains to be established. While some reports confirm their Néel helicity in chiral multilayers,^[^ ^3^, ^18^ ^]^ others have reported a considerable Bloch component with a layer‐ and material‐dependent magnitude.^[^ ^19^, ^20^ ^]^ Meanwhile, experimental investigations of the field evolution of their size have focused narrowly on “bubble skyrmions.”^[^ ^3^ ^]^ Even theoretical efforts, while fully exploring isolated skyrmions, are yet to examine the rich interactions between skyrmions and stripes.^[^ ^21^, ^22^ ^]^ Finally, while morphological transitions from stripes to skyrmions have been demonstrated,^[^ ^14^, ^23^ ^]^ the thermodynamic mechanism of skyrmion formation is not understood. Elucidating the microscopic origin of these attributes necessitates a multi‐modal investigation of chiral spin textures with varying magnetic interactions on a single material platform.

Much of the character of chiral spin textures can be tuned by a single material parameter, $\kappa =\pi D/4\sqrt{AK_{\text{eff}}}$, where *D* is the interfacial Dzyaloshinskii–Moriya interaction (iDMI), *A* is the exchange stiffness and *K*_eff_ is the effective uniaxial anisotropy.^[^ ^24^, ^25^, ^26^ ^]^ Within a simple analytical model without assumption of sample symmetry, *κ* is associated with the DW energy—*κ* > 1 implies that DW energy density is negative.^[^ ^24^, ^27^ ^]^ Here, we investigate the emergent characteristics of chiral spin textures over a wide range of *κ* on a tunable Co/Pt‐based multilayer platform. Exploiting the complementary sensitivity of Lorentz transmission electron microscopy (LTEM) and magnetic transmission soft X‐ray microscopy (MTXM), to textural characteristics, we elucidate the evolution of DW helicity, domain compressibility, and skyrmion formation mechanism with increasing *κ*. In conjunction with micromagnetic simulations and an analytical model, we establish a microscopic framework for spin texture character in multilayer films.

Our work is performed at RT using Co/Pt‐based multilayer stacks with out‐of‐plane (OP) anisotropy, which are established hosts of magnetic textures.^[^ ^28^ ^]^ While symmetric stacks have negligible total iDMI, asymmetric stacks, such as (Ir or Ta)/Co/Pt can have sizable iDMI (*D* > 1 mJ m^−2^)—relevant to chiral magnetic textures.^[^ ^1^, ^2^, ^3^ ^]^ The inclusion of Fe—as in Ir/Fe/Co/Pt stacks—enhances the iDMI, while *D* and *K*_eff_ can be smoothly modulated by Fe and Co thicknesses.^[^ ^4^ ^]^ Here we study four samples, each comprising 1 nm thick FM layers—identified by their **Fe(x)/Co(y)** composition (**Table** **1**)—wherein the active stack is repeated 14 times to optimize full‐field magnetic contrast. Interfacial interactions are progressively introduced and quantified using established techniques,^[^ ^1^, ^2^, ^4^ ^]^ with the estimated iDMI (*D*_est_) varying over 0–2 mJ m^−2^ and *K*_eff_ over 0.08–0.70 MJ m^−3^ (Experimental Section and Section S1, Supporting Information). Notably, the *D*_est_ determined for the 14‐stack multilayers studied here are in line with measured values on corresponding single stacks from Brillouin light scattering (BLS) experiments.^[^ ^29^ ^]^ Consequently, *κ* varies over 0–1.5, and provides the requisite range for mapping magnetic texture evolution.

**Table 1:** Sample compositions. List of multilayer samples used in this work, with layer thickness in angstroms in parentheses (see Experimental Section and Section S1, Supporting Information for full‐stack details). Corresponding magnetic properties are listed: effective anisotropy (***K*_eff_** MJ m^−3^), estimated iDMI (***D*_est_** mJ m^−2^) and the stability parameter ***κ***. The samples are henceforth referred to by their acronym

**Acronym**	**Stack composition [Å]**	$K_{\text{eff}}$	$D_{\text{est}}$	κ
**^S^Co(10)**	[Pt(10)/Co(10)/Pt(10)]_14_	0.68	0	0
**Fe(0)/Co(10)**	[Ir(10)/Co(10)/Pt(10)]_14_	0.47	1.3	0.3
**Fe(2)/Co(8)**	[Ir(10)/Fe(2)/Co(8)/Pt(10)]_14_	0.22	1.8	0.9
**Fe(3)/Co(7)**	[Ir(10)/Fe(3)/Co(7)/Pt(10)]_14_	0.08	2.0	1.5

## 2 DW Helicity

**Figure 1:**
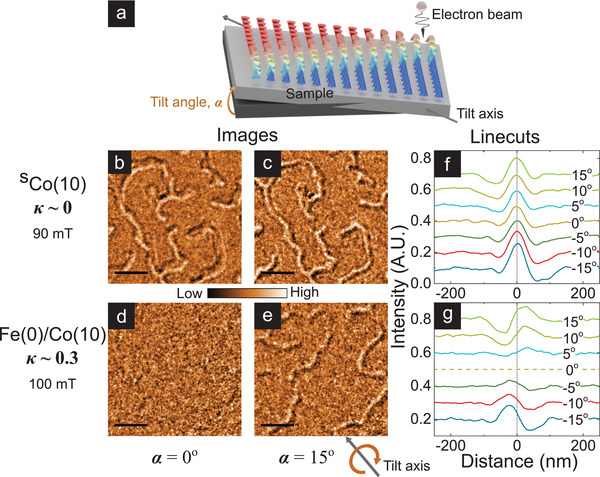
DW helicity from tilt‐dependent LTEM imaging. a) Schematic diagram of LTEM imaging geometry with sample (illustrated here with a Néel DW) tilted at angle α with respect to the plane normal to the electron beam. The tilt axis is shown below (e). b–e) Representative LTEM images (scale bar: 0.5 µm) acquired on samples ^S^Co(10) (b,c) and Fe(0)/Co(10) (d,e) at µ_0_ *H* = 90 and 100 mT respectively for α = 0° (b) and (d) and α = 15° (c) and (e), with −2 mm defocus. f,g) Average cross‐sectional linecuts across domains detected in LTEM images of ^S^Co(10) (f) and Fe(0)/Co(10) (g), with α varied over ±15°. Each curve represents the average of ≈1000 linecuts extracted from domains imaged over a 5 µm field‐of‐view using an automated recipe. Dashed vertical lines mark the domain center.

The introduction of iDMI should lead to a measurable change in DW helicity of skyrmion textures.^[^ ^7^, ^30^ ^]^ LTEM imaging—wherein magnetic contrast results from the magnetization curl parallel to the electron beam—is particularly sensitive to such changes. For normal beam incidence (zero tilt), a pair of homochiral Bloch DWs should express symmetric contrast about their center, while Néel DWs should exhibit no contrast as their curl is perpendicular to the beam.^[^ ^31^ ^]^ Meanwhile, the positions of Néel DWs can be deduced by tilting the sample (**Figure** **1**a), whereupon antisymmetric domain contrast can be observed.^[^ ^18^ ^]^ To visualize this evolution, we perform tilt‐dependent LTEM imaging with samples deposited on SiO_*x*_ membranes (see Experimental Section). For ease of analysis, we use OP magnetic fields (µ_0_ *H*) large enough to ensure adjacency of pairs of DWs (i.e., thin domains). Artifacts due to granularity and membrane waviness are mitigated using a recipe that extracts ≈1000 linecuts across domains imaged over a 5 µm field‐of‐view (see Section S2, Supporting Information).

Figure 1 shows stark differences in tilt‐dependent LTEM results for ^S^Co(10) and Fe(0)/Co(10). First, ^S^Co(10) (*D*_est_ ≃ 0, *κ* ≃ 0) shows strong, symmetric contrast about the domain center at zero tilt (Figure 1b), with a small antisymmetric component at finite tilt angle (Figure 1c,f). This is consistent with Bloch DWs expected for symmetric stacks.^[^ ^32^, ^33^, ^34^ ^]^ Micromagnetic simulations performed with ^S^Co(10) parameters (see Experimental Section) suggest that the Bloch DWs are achiral, that is, lack fixed handedness (see Section S4, Supporting Information). In comparison, Fe(0)/Co(10) (*D*_est_ ≃ 1.3 mJ m^−2^, *κ* ≃ 0.3) shows no contrast at zero tilt (Figure 1d). Contrast at finite tilt is consistently antisymmetric—and its amplitude increases with tilt angle (Figure 1e,g)—consistent with Néel DWs. The lack of measurable symmetric contrast in Figure 1g suggests that any Bloch component—for example, due to layer‐dependent chirality^[^ ^19^, ^20^ ^]^—is negligibly small.^[^ ^34^ ^]^ Micromagnetic simulations for Fe(0)/Co(10) also reflect the limited influence of such layer‐dependent variations, which are further suppressed if moderate interlayer exchange coupling is included (see Section S4, Supporting Information). Finally, similar experiments on *κ* ≳ 1 samples produce results fully consistent with Fe(0)/Co(10) (see Section S3, Supporting Information). These results indicate that chiral interactions in the *κ* ≈ 0.3 sample are sufficiently large to transform achiral Bloch textures (*κ* ≃ 0) to homochiral Néel textures.^[^ ^27^ ^]^

## 3 Domain Compressibility

Having established DW helicity evolution, we turn to domain characteristics—which evolve with OP field in addition to magnetic interactions.^[^ ^1^, ^2^, ^4^ ^]^ Both stripes and skyrmions can be collectively characterized by a single length scale, *W*, defined as domain width of stripes and diameter of skyrmions of the minority polarization. Notably, the field‐induced variation of *W*, or d*W*/d*H*—termed as domain compressibility^[^ ^35^ ^]^—should also evolve with *κ*.^[^ ^4^, ^25^, ^36^ ^]^ MTXM imaging—wherein magnetic circular dichroic contrast is proportional to local OP magnetization^[^ ^37^ ^]^—is well‐suited to measure *W*. Therefore, we performed MTXM imaging with varying OP field using samples deposited on Si_3_N_4_ membranes, complemented by micromagnetic simulations (see Experimental Section). *W* was determined as an averaged quantity over the full field‐of‐view of about 5 µm using an automated recipe to mitigate granularity effects (see Section S2, Supporting Information). The identity of the minority polarization flips at the coercive field, resulting in a sharp kink in *W*.

**Figure 2:**
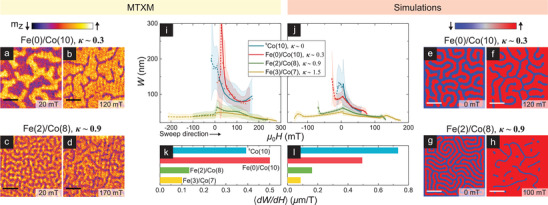
Domain width field evolution: MTXM imaging and simulations. a–d) MTXM images of samples Fe(0)/Co(10) (*κ* ≈ 0.3: a,b) and Fe(2)/Co(8) (*κ* ≈ 0.9, c,d) – showing domain evolution from near‐zero (a,c) to near‐saturation (b,d) fields (scalebar: 0.5 µm). e–h) Simulated magnetization images for magnetic parameters consistent with Fe(0)/Co(10) (e,f) and Fe(2)/Co(8) (g,h)—showing the corresponding evolution from zero (e,g) to higher fields (f,h). i,j) Average minority polarization domain width, *W*(*H*), with varying magnetic field from MTXM experiments (i) and simulations (j) on all four samples studied in this work. Each *W*(*H*) data point is a full field‐of‐view mean (shaded band is the standard deviation) determined using an automated recipe (See Section S2, Supporting Information). Solid and dashed lines, guides to the eye, represent magnetization in the negative and positive vertical direction respectively. k,l) Average magnitude of compressibility, 〈d*W*/d*H*〉, for the four samples from experiments (k) and simulations (l), corresponding to the average gradients in (i) and (j) respectively for µ_0_ *H* > 0.

**Figure** **2** shows MTXM (a–d,i,k) and simulation results (e–h,j,l) of *W*(*H*) across samples with varying *κ*. On one hand, for *κ* ≪ 1—illustrated for Fe(0)/Co(10) (*κ* ≈ 0.3, Figure 2a,b,i)—*W* shrinks rapidly with field (〈d*W*/d*H*〉 ≈ 0.5 µm T^−1^, Figure 2k). Such highly compressible behavior is well reproduced by simulations (Figure 2e,f). One difference from experiments is the relative order of 〈d*W*/d*H*〉 for ^S^Co(10) and Fe(0)/Co(10). The likely source of this discrepancy is the domain nucleation field, which affects domain compressibility. In low *κ* samples such as ^S^Co(10) and Fe(0)/Co(10), domain nucleation may be dominated by extrinsic factors such as grains^[^ ^38^ ^]^ that are not accounted for in our simulations. Nevertheless, the *W*(*H*) trend of chiral Néel textures (Fe(0)/Co(10)) is remarkably similar to achiral Bloch textures (^S^Co(10)),^[^ ^39^ ^]^ Figure 2i,j). This suggests that domain compressibility is largely independent of DW helicity. On the other hand, for *κ* ≳ 1 – shown for Fe(2)/Co(8) (κ ≈ 0.9, Figure 2c,d,i) – the *W*(*H*) variation is much reduced (〈d*W*/d*H*〉 ≈ 0.1 µm T^−1^, Figure 2k).^[^ ^40^ ^]^ Similarly rigid or incompressible behavior is seen for Fe(3)/Co(7) (*κ* ≈ 1.5), albeit at reduced *W*, and in the analysis of LTEM images (see Section S3, Supporting Information). Finally, simulated trends for *κ* ≳ 1 are also in line with these results (Figure 2g,h,j,l), suggesting that the contrast may be understood within a micromagnetic energy framework.

**Figure 3:**
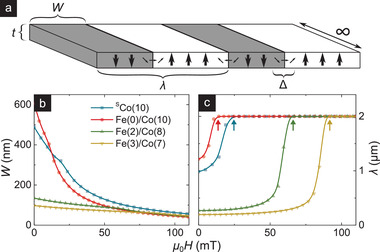
1D model for domain compressibility evolution. a) Schematic of the simplified analytical model of 1D periodic domains used to interpret the observed domain compressibility. Minority domains of DW width Δ, total width *W*, and period λ are considered within an infinitely long magnetic slab of thickness *t* and breadth 2 µm. b,c) Field evolution of domain width, *W*(*H*) (b, c.f., Figure 2i,j) and period, λ(*H*) (c), obtained from the 1D model (details in text) for magnetic parameters consistent with the samples of interest. Arrows in (c) mark the saturation field, *H*_S_, predicted by the model.

To elucidate the compressibility evolution, we use an analytical model of 1D periodic domains within an infinite magnetic slab of thickness *t*, domain period λ, and DW width Δ (**Figure** **3**a). This model is chosen because it incorporates interactions between neighboring stripes, which appears relevant to the compressibility phenomenon. The total energy density is given by^[^ ^21^ ^]^

(1) $$\begin{array}{ccc} \varepsilon_{\text{tot}} & = & \frac{2}{\lambda }\left[\frac{2A}{\Delta }+2K_{u}\Delta +\pi D\sin\psi \right]+\varepsilon_{\text{d,s}}+\varepsilon_{\text{d,v}}\\ & & -\;M_{s}\left(1-\frac{2W}{\lambda }\right)B_{z}, \end{array}$$

where the magnetostatic energy densities due to surface (ε_d, s_) and volume charges (ε_d, v_) are:

(2) $$\begin{array}{ccc} \varepsilon_{\text{d,s}} & = & \frac{\mu_{0}M_{s}^{2}}{2}{\left(1-\frac{2W}{\lambda }\right)}^{2}\\ & & +\;\frac{2\pi \mu_{0}M_{s}^{2}\Delta^{2}}{\lambda t}\sum_{n=1}^{\infty }\frac{\sin^{2}\frac{\pi nW}{\lambda }}{\sinh^{2}\frac{\pi^{2}n\Delta }{\lambda }}\frac{1-\exp\left(-\frac{2\pi nt}{\lambda }\right)}{n},\text{and} \end{array}$$

(3) $$\begin{array}{ccc} \varepsilon_{\text{d,v}} & = & \frac{2\pi \mu_{0}M_{s}^{2}\Delta^{2}\sin^{2}\psi }{\lambda t}\\ & & \times \;\sum_{n=1}^{\infty }\frac{\sin^{2}\frac{\pi nW}{\lambda }}{\cosh^{2}\frac{\pi^{2}n\Delta }{\lambda }}\frac{\exp\left(-\frac{2\pi nt}{\lambda }\right)+\frac{2\pi nt}{\lambda }-1}{n}. \end{array}$$

The field evolutions of *W* and λ (Figure 3b,c) are obtained by numerically minimizing ε_tot_ with respect to λ, *W*, and Δ. Notably, the 1D model reproduces *κ* dependence of compressibility found in experiments (Figure 2i) and simulations (Figure 2j): domains are highly compressible for *κ* ≪ 1, and relatively incompressible for *κ* ≳ 1. Furthermore, it offers a physical explanation for the compressibility evolution when viewed in conjunction with λ(*H*). The latter is indicative of the saturation field, *H*_S_ (arrows in Figure 3c), and the domain density. For *κ* ≳ 1, wherein *H*_S_ is higher (see Section S1, Supporting Information), domain nucleation occurs just below *H*_S_ with smaller size (Figure 3b) and very close proximity (Figure 3c). The latter ensures mutual confinement of domains, limiting the expansion of *W* with reducing *H*. Therefore, as *H* is increased from zero, *κ* ≳ 1 domains have limited latitude for compression, and *W*(*H*) is nearly constant—expectedly near the lower cut‐off (*W* ≈ Δ). The converse argument holds for *κ* ≪ 1 domains, which explains their highly compressible *W*(*H*) behavior.

The marked variation of domain compressibility with *κ*, its direct experimental accessibility, and consistency with grain‐free simulations and the 1D model, establish compressibility as an important classifier of skyrmions (and stripes). Compressibility incorporates energetic considerations underlying a theoretically proposed “minimum skyrmion size” metric for isolated skyrmions^[^ ^21^ ^]^ (details in Section S6, Supporting Information), while also being relatively robust to material complexities such as grains and defects compared to the size of isolated skyrmions.^[^ ^21^, ^41^ ^]^ Therefore, it can serve as a useful means to experimentally differentiate highly compressible “bubble” skyrmions from relatively incompressible “compact” skyrmions. Meanwhile, the remarkable difference in compressibility between samples with *κ* ≪ 1 and *κ* ≳ 1 demonstrates the importance of considering interactions between skyrmion textures within theoretical models. Further, it hints at the possibility of using effective fields, generated by material, geometric, or external means,^[^ ^36^, ^42^, ^43^ ^]^ to tune the size and morphology of stabilized spin textures.

## 4 Skyrmion Formation Mechanism

**Figure 4:**
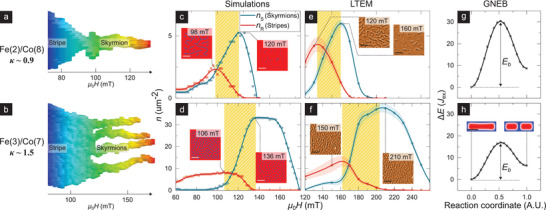
Evolution of skyrmion formation mechanism. a,b) Simulated field evolution of a prototypical stripe after its separation from the labyrinthine state for a) Fe(2)/Co(8) (*κ* ≈ 0.9) and b) Fe(3)/Co(7) (*κ* ≈ 1.5) parameters. The stripe is identified and isolated in real‐space for each field slice and stacked horizontally to form a 3D structure, which is in turn reoriented with the length of the stripe along the vertical and field axis along the horizontal (details in Section S4, Supporting Information). c–f) Field evolution of the density of skyrmions (*n*_S_, teal) and stripes (*n*_R_, red), as extracted from simulations (c,d) and LTEM imaging (e,f) for samples Fe(2)/Co(8) (c,e) and Fe(3)/Co(7) respectively (procedural details in Section S2, Supporting Information). Highlighted regions denote field ranges corresponding to marked stripe to skyrmion transitions (c.f., a,b). Inset: Simulation and LTEM images immediately before and after stripe‐to‐skyrmion transitions (scalebar: 0.5 µm). g,h) Energy profile (in units of Heisenberg exchange *J*_ex_) governing the fission of a stripe for Fe(2)/Co(8) (*κ* ≈ 0.9) and Fe(3)/Co(7) (*κ* ≈ 1.5) respectively calculated using GNEB method (details in Section S5, Supporting Information). *E*_b_ denotes the energy barrier for the fission process, inset of (h) depicts the stripe before and after fission (scalebar: 0.1 µm).

While skyrmions are known to emerge from stripes with increasing field, the transition may involve one or more mechanisms or paths. Notably, *κ*, which determines DW stability, is also expected to affect this stripe‐to‐skyrmion transition. First, we visually examine the *κ*‐variation of this transition by tracking the simulated field evolution of a prototypical magnetic stripe (details in Section S4, Supporting Information). We see for *κ* ≈ 0.9 (**Figure** **4**a) that the stripe shrinks smoothly with field, and eventually turns into a single skyrmion. Meanwhile, for *κ* ≈ 1.5 (Figure 4b), the stripe abruptly fissions into four distinct skyrmions at a characteristic field.^[^ ^44^ ^]^ These two mechanisms should result in contrasting textural field evolutions that should be detectable in our experiments. Therefore, we statistically examine the field evolution of stripes and skyrmions—distinguished in images by their circularity (See Section S2, Supporting Information). Here, we choose LTEM imaging, as it enables a clearer distinction between skyrmions and stripes (See Section S2, Supporting Information).

Figure 4c–f present the field evolution of densities of skyrmions (*n*_S_) and stripes (*n*_R_) from LTEM and simulations for samples Fe(2)/Co(8) and Fe(3)/Co(7). For each case, highlighted regions at intermediate fields—spanning from *n*_R_ peak to *n*_S_ peak–indicate stripe‐to‐skyrmion transitions, and exhibit contrasting trends. For Fe(2)/Co(8) (*κ* ≈ 0.9, Figure 4c,e) the decrease in *n*_R_ (≈2–3 µm^−2^) corresponds to a one‐to‐one increase in *n*_S_ (≈2–3 µm^−2^). This is consistent with the shrinking of one stripe to one skyrmion, thereby resulting in isolated skyrmions (Figure 4c,e: inset). In contrast, for Fe(3)/Co(7) (*κ* ≈ 1.5, Figure 4d,f) the decrease in *n*_R_ (≈7–8 µm^−2^) coincides with a fourfold increase in *n*_S_ (≈30 µm^−2^). This is in line with the fission of one stripe into ≈4 skyrmions on average and generates a dense skyrmion lattice (Figure 4d,f: inset).^[^ ^44^ ^]^ Thus, we have empirically observed the increased favorability of fission with increasing *κ* (0.9 to 1.5).

The above observation may be understood from kinetic considerations. The fission of a stripe involves a change in topology and hence should be protected by an energy barrier. To examine the evolution of the barrier height (*E*_b_) with *κ*, we perform geodesic nudged elastic band (GNEB) calculations for Fe(2)/Co(8) and Fe(3)/Co(7) (Figure 4g,h, details in Section S5, Supporting Information). We found that *E*_b_ for Fe(2)/Co(8) is 40% greater than that for Fe(3)/Co(7). Assuming that entropic effects are comparable across the two compositions,^[^ ^45^ ^]^ it follows that fission will be greatly suppressed in Fe(2)/Co(8) relative to Fe(3)/Co(7). The suppression of fission in Fe(2)/Co(8) will then require stripes to instead smoothly shrink into skyrmions.

## 5 Outlook

**Figure 5:**
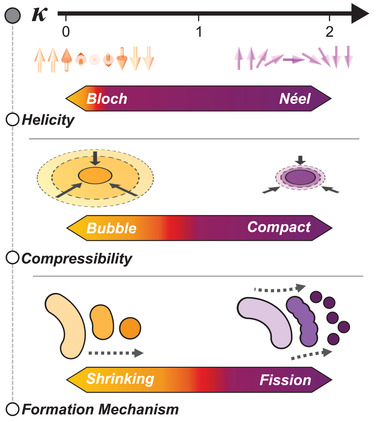
*κ*‐driven evolution of skyrmion character. Overview of the evolution of multilayer skyrmion characteristics with increasing *κ* as seen across the samples studied in this work. This includes the change of DW helicity from Bloch to Néel (top), domain compressibility from bubble to compact (middle), and skyrmion formation mechanism from shrinking to fission of stripes (bottom).

In summary, we have elucidated transitions in three critical characteristics of chiral spin textures. As shown in **Figure** **5**, these characteristics systematically evolve with *κ*—the material parameter determining chiral DW stability. First, as *κ* increases measurably from zero, the DW helicity transitions from achiral Bloch to chiral Néel‐type. Next, as *κ* approaches unity, the domain compressibility is drastically reduced, transforming “bubble” skyrmions into “compact” skyrmions. Finally, for *κ* > 1, the skyrmion formation mechanism evolves from shrinking to fission of stripes, resulting in the proliferation of compact Néel skyrmions for *κ* > 1.

Our findings—established on a single tunable material platform—provide several valuable insights toward understanding the observed phenomenology of chiral spin textures, notably skyrmions. First, we have shown that a small but finite *κ* (≈0.3) enables the formation of Néel DWs with fixed chirality, with no evidence of a Bloch component even for a 14‐repeat stack. Next, we have established the compressibility of domains as a robust experimental metric to differentiate bubble and compact skyrmions. Finally, we have shown that the preference for one of two distinct skyrmion formation mechanisms—shrinking and fission—may explain the observation of isolated skyrmions^[^ ^1^, ^13^ ^]^ in some materials and dense skyrmion lattices^[^ ^4^, ^9^ ^]^ in others.

These insights provide a timely roadmap to inform stack design for skyrmionic applications—particularly in device architectures that rely on ensembles of chiral spin textures rather than on sparse, isolated skyrmions. For example, selecting a stack with 0 < *κ* ≪ 1, hosting highly compressible domains, will enable dynamic tuning of the spin texture morphology with temporal variation of applied fields. Conversely, if the application requires control of the topology of textures, a *κ* ≫ 1 stack, enabling fission‐driven skyrmion formation, would be a better fit. Spanning the physics of stripes and skyrmions, our work provides a springboard for their use as “skyrmion fabrics” for applications in unconventional computing.^[^ ^16^ ^]^

## 6 Experimental Section

### Sample Fabrication

Multilayer films, comprising Ta(40)/Pt(50)/**[HM(10)/Fe(x)/Co(y)/HM(10)]14**/Pt(20) (HM: heavy metal, number in parentheses indicates thickness in angstroms), were deposited by DC magnetron sputtering at room temperature using a Chiron UHV system manufactured by Bestec GmbH (base pressure: 10^−8^ Torr). Four samples were studied in this work whose active stack compositions (bolded above) are listed in Table 1. To enable direct comparison between different techniques used in this work, the films were simultaneously deposited for magnetometry on thermally oxidized 100 nm Si wafer substrates, for LTEM on 20 nm‐thick SiO_2_ membrane window grids from SPI Supplies, and for MTXM on 50–200 nm thick Si_3_N_4_ membranes from Silson. Magnetometry measurements were performed using an EZ11 vibrating sample magnetometer (VSM) made by MicroSense. The magnetic parameters: *M*_*S*_, *K*_eff_, *A*_est_, and *D*_est_ were obtained using protocols consistent with literature,^[^ ^1^, ^2^, ^4^, ^36^, ^46^ ^]^ and are detailed in Section S1, Supporting Information.

### Lorentz TEM Experiments

Lorentz transmission electron microscopy (LTEM) experiments were performed using an FEI Titan S/TEM operated in Lorentz Fresnel mode at 300 kV. A dedicated Lorentz lens for focusing the electron beam was used at a defocus of −2 mm. Meanwhile, the objective lens located at the sample position was switched off for imaging acquisition under field‐free conditions, or excited to different strengths to apply out‐of‐plane magnetic fields (−300 mT to +2 T) for in situ studies of magnetic texture evolution.

### MTXM Experiments

Full‐field MTXM imaging experiments were performed using circularly polarized soft X‐rays at the advanced light source (XM‐1 BL 6.1.2), using the Co L3 edge (≈778 eV) with OP sample geometry.^[^ ^37^ ^]^ OP magnetic fields were applied using an electromagnet, and a pair of horse‐shoe poles were used to guide the generated flux.

### Micromagnetic Simulations

Micromagnetic simulations were performed using MuMax3 to interpret the field evolution of the 14 repeat multilayer stacks.^[^ ^47^ ^]^ The simulation field‐of‐view used was 2 µm × 2 µm, and the cell size was kept to 4 nm × 4 nm × 3 nm, which was below the exchange length for all samples. The effective medium approximation was used with one layer per stack repetition to account for memory constraints.^[^ ^2^ ^]^ Hysteresis loops were simulated using protocols described in ref. [47]

### Image Analysis

Custom‐written Python scripts were used for the quantitative analysis of magnetic microscopy images. These scripts comprised routines for image filtering and binarization followed by domain characterization and statistics using standard methods in the scikit‐image library.^[^ ^48^ ^]^ The analysis procedures were detailed in Section S2, Supporting Information.

## Conflict of Interest

The authors declare no conflict of interest.

## Supporting information

Supporting Information

Supplemental Video 1

Supplemental Video 2

## Data Availability

Data available on request from the authors
