# Supplementary material for: Unveiling the Emergent Traits of Chiral Spin Textures in Magnetic Multilayers
Source: Adv Sci (Weinh). 2022 Jan 2;9(6):2103978. doi: 10.1002/advs.202103978 (PMC8867163; doi:10.1002/advs.202103978)
Supplement: Supplementary file 1 — Supporting Information [file ADVS-9-2103978-s002.pdf]

ADVANCED SCIENCE
Open Access

Supporting Information

for *Adv. Sci.,* DOI: 10.1002/advs.202103978

Unveiling the emergent traits of chiral spin textures in magnetic multilayers

Xiaoye Chen, Ming Lin, Jian Feng Kong,* Hui Ru Tan, Anthony K.C. Tan, Soong-Geun Je, Hang Khume Tan, Khoong Hong Khoo, Mi-Young Im, and Anjan Soumyanarayanan*

Supporting Information for

Unveiling the emergent traits of chiral spin textures in magnetic multilayers

Xiaoye Chen,[1,2] Ming Lin,[1,*] Jian Feng Kong,[3,*] Hui Ru Tan,[1] Anthony K.C. Tan,[2] Soong-Geun Je,[4] Hang Khume Tan,[1,2] Khoong Hong Khoo,[3] Mi-Young Im,[4] and Anjan Soumyanarayanan[1,2,5,†]

[1] *Institute of Materials Research & Engineering, Agency for Science, Technology & Research (A*STAR), 138634 Singapore*

[2] *Data Storage Institute, Agency for Science, Technology & Research (A*STAR), 138634 Singapore*

[3] *Institute of High Performance Computing, Agency for Science, Technology & Research (A*STAR), 138632 Singapore*

[4] *Center for X-ray Optics, Lawrence Berkeley National Laboratory, Berkeley, California 94720, USA*

[5] *Physics Department, National University of Singapore (NUS), 117551 Singapore*

S1. Magnetic Properties of Multilayer Samples

Magnetic Properties. The magnetic properties of the samples studied in this work are tabulated in Table S1 and plotted in Figure S1. The saturation magnetization, M_s, and effective anisotropy, K_{eff}, were determined from magnetometry measurements (see Figure S2a-d). Following procedures established in previous works[1], the exchange stiffness, A_{est}, was estimated with density functional theory (DFT)[2]. Meanwhile, the iDMI value, D_{est} was estimated by comparing the periodicity of micromagnetic simulations at zero field with that from magnetic force microscopy (MFM) images (see Figure S2q-t)[1,3,4]. Notably, the values A_{est} and D_{est} for the 14x multilayers studied in this work are in good agreement with values obtained for corresponding 1x films from Brillouin light scattering (BLS) experiments[2]. To check if a small iDMI affects the simulation of the ^{S}Co(10) stack, we performed additional hysteresis loop simulations with iDMI of 0.1 mJ/m^2[2] and found the results to be virtually identical to our original zero iDMI simulations.

Magnetometry and magnetic microscopy. In Figure S2, we show the magnetization hysteresis loops, $M(H)$, with applied field $\mu_0 H$ in the in-plane (IP) and out-of-plane (OP) directions. We also show an overview of LTEM, MTXM and micromagnetic simulations for the four samples at zero field or low fields, in the labyrinthine stripe state. The

Acronym	Stack Composition	M_s (MA/m)	K_{eff} (MJ/m^3)	D_{est} (mJ/m^2)	A_{est} (pJ/m)	κ
**^{S}Co(10)**	$[Pt(10)/Co(10)/Pt(10)]_{14}$	1.18	0.68	0	17.3	0
**Fe(0)/Co(10)**	$[Ir(10)/Co(10)/Pt(10)]_{14}$	0.88	0.47	1.3	17.8	0.3
**Fe(2)/Co(8)**	$[Ir(10)/Fe(2)/Co(8)/Pt(10)]_{14}$	0.93	0.22	1.8	12.8	0.9
**Fe(3)/Co(7)**	$[Ir(10)/Fe(3)/Co(7)/Pt(10)]_{14}$	0.96	0.08	2.0	13.6	1.5

Table S1. **Magnetic properties of multilayer samples.** List of multilayer samples used in this work, with layer thickness in angstroms in parentheses. The magnetic properties – saturation magnetization (M_s), effective magnetic anisotropy (K_{eff}), estimated iDMI (D_{est}), estimated exchange stiffness (A_{est}) as well as the stability parameter, κ – are tabulated.

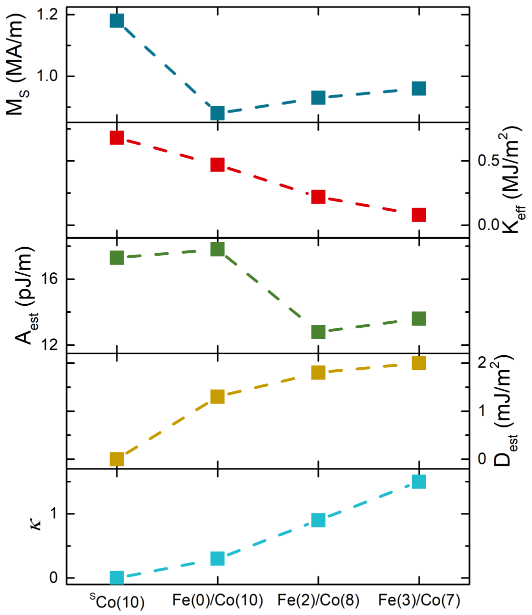

Figure S1. **Magnetic properties of multilayer samples.** The trendlines of M_s, K_{eff}, A_{est}, D_{est} and κ across samples. The samples are arranged in order of increasing κ.

magnetic morphology captured by the two experimental imaging techniques is consistent. The simulation results are also largely in agreement with the experimental images.

S2. Image analysis

Basic image processing. The analysis of LTEM and MTXM images used in this work was performed with custom-written Python code. To remove low spatial frequency background, a duplicate convolved with a large Gaussian kernel was subtracted from the original image. To reduce the high spatial frequency noise while preserving as much information as possible, a small median filter was then applied. In cases where binarization was required, the threshold was automatically selected using the Otsu algorithm. To remove further noise, if any, small regions below a certain areal threshold were disregarded.

Domain width analysis. Manuscript Fig. 2 shows the averaged domain width from MTXM images. To obtain this result, background removal, denoising and binarization were performed as described above. Next, a Euclidean distance transform – which yields the shortest distance from the domain edge to the medial axis – was applied on the binarized image using built-in functions in the scikit-image library[5]. Finally, the average domain width was extracted as twice the average distance to the medial axis.

Linecut analysis. Manuscript Fig. 1 shows linecuts across stripe domains at fixed intervals from LTEM images. To obtain these linecuts, a skeletonization operation was enacted on the LTEM images after the basic image processing and binarization described above. Next, all foreground pixels connected to more than two other pixels were removed. This ensured that there were no branches and that all regions, defined as foreground pixels that are 4-connected, are linear. Each region was then fitted to a spline, and the normal to the spline was computed. Finally, linecuts were acquired from the original, unprocessed LTEM image using methods in scikit-image library, at regular intervals on the spline. Linecuts exceeding the boundaries of the image were excluded. The position and the direction of the algorithmically chosen linecuts are shown in Figure S3. The acquired linecuts were further binned by angle, and only those within 10° from the tilt axis were included in calculating the averaged output.

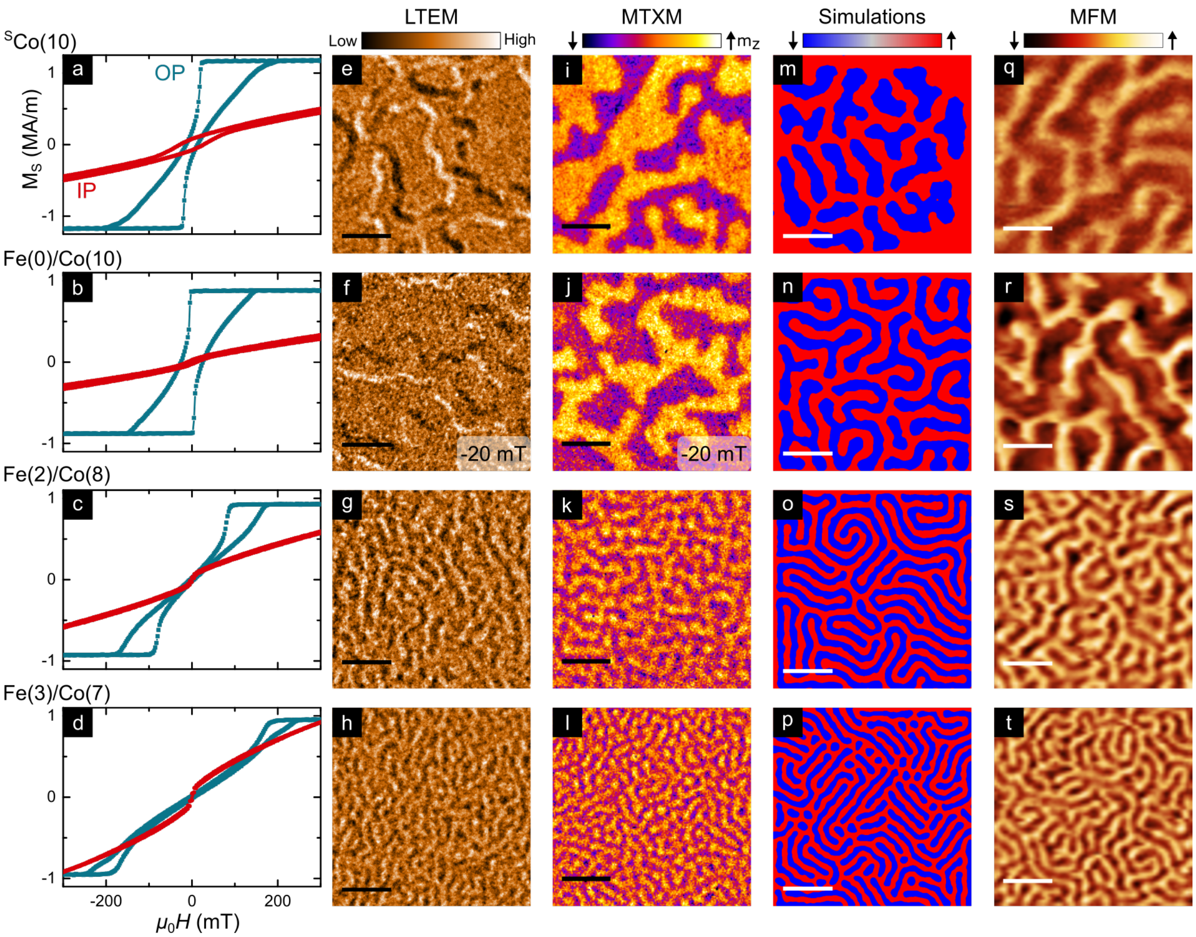

Figure S2. **Overview of magnetometry and magnetic imaging. (a-d)** Magnetization hysteresis loops for in-plane (IP, red) and out-of-plane (OP, teal) orientations of applied field, $\mu_0 H$, across all four samples. Equilibrium magnetic configurations of all four samples as observed in **(e-h)** LTEM images ($\alpha = 20°$ and -2 mm defocus), **(i-l)** MTXM images, **(m-p)** micromagnetic simulations and **(q-t)** MFM images. All images are at the same scale and are acquired at zero-field apart than (f,j), which are at -20 mT. Scalebar: 0.5 μm.

For analysis of LTEM images acquired at varying tilt angles, an additional preprocessing step was required. The sequence of images had to be aligned to the first image, as the latter was used to determine the position of each linecut. The image alignment was done manually with ImageJ by selecting about 15 corresponding landmarks on each image and calculating an affine transformation to map each image in the tilt sequence to the first image. Manuscript Fig. 1 does not show the linecut for zero tilt of Fe(0)/Co(10). This is because, in the absence of visible magnetic textures at zero tilt, the alignment landmarks for that image could not be located. Similar image sequence alignment issues precluded the use of this method for analyzing Fe(3)/Co(7) images (see § S3).

Domain counting. Manuscript Fig. 4 details the statistics of skyrmion and stripe number densities with varying magnetic field, which were obtained as follows. For micromagnetic simulations (manuscript Fig. 4c,d), algorithmic counting gave reliable results after denoising and binarization due to the high signal-to-noise ratio (SNR) of domains in simulated images. Domains that exceeded a visually determined circularity threshold of 0.65, were classified as skyrmions, while the remainder otherwise, were counted as stripes. Here, circularity is defined as $4\pi A/P^2$, where A and P are the area and perimeter of the domain respectively. For LTEM, attempts to use such algorithmic counting of stripes and skyrmions did not give results consistent with visual inspection. Hence, we visually identified every texture in LTEM images as a skyrmion or stripe. A typical LTEM image of Fe(2)/Co(8) with skyrmions and stripes

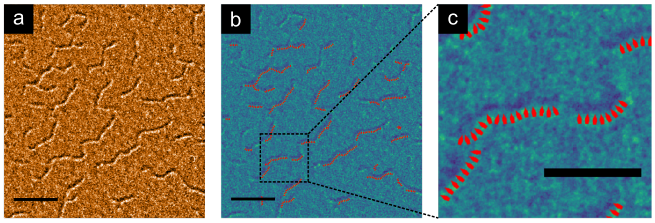

Figure S3. **Illustration of linecut analysis for LTEM images.** An illustration of the intermediate processing steps used to obtain linecuts from LTEM images shown in manuscript Fig. 1f-g. **(a)** Raw LTEM image of Fe(0)/Co(10) at −100 mT, $\alpha = -20°$ and −2 mm defocus (scalebar: 1 um). **(b)** Algorithmically determined linecuts (red lines) overlaid on the image in (a). **(c)** A zoomed-in view of (b), showing individual linecuts, as indicated by arrows (scalebar: 0.5 um).

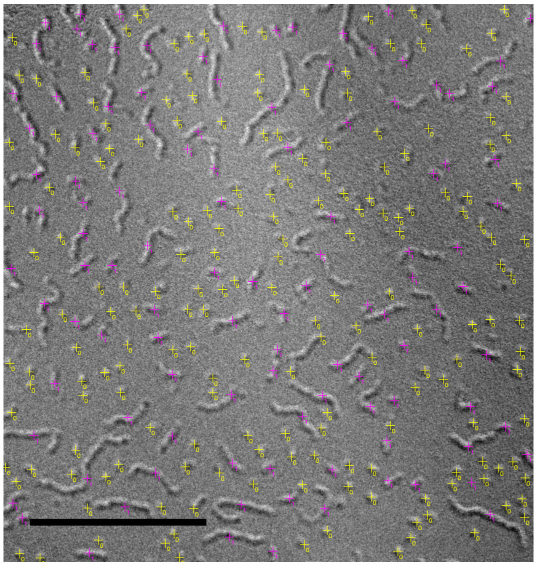

Figure S4. **Identification of textures in LTEM images.** A representative LTEM image of Fe(2)/Co(8) at 150 mT, $\alpha = 20°$ and −2 mm defocus (scalebar: 1 um) used to demonstrate the counting of observed skyrmions and stripes. Skyrmions are indicated with yellow crosses, and stripes with pink crosses.

identified as above is shown in Figure S4.

S3. LTEM Imaging

LTEM contrast simulation. In this work, spatial evolution of LTEM contrast of textures has been used to quantify their helicity. As LTEM contrast is not intuitive to non-experts, we show in Figure S5 the simulated LTEM contrast – generated using an open-source software, MALTS[6] – for an idealized magnetic stripe with Bloch and Néel DWs. Also shown are the linecuts perpendicular to the domain for various tilt angles (c.f. manuscript Fig. 1f-g). At zero tilt, the Bloch DW pair produces contrast symmetric about the center of the stripe, whereas the Néel DW pair give zero contrast. In both cases, an additional antisymmetric contrast is present for finite tilt.

Additional LTEM data for DW helicity. In manuscript Fig. 1, we have shown tilt-dependent LTEM data for ^{S}Co(10), confirming Bloch helicity of its DWs, and for Fe(0)/Co(10) – wherein DWs have Néel helicity. In Figure S6, we show for comparison LTEM data for Fe(2)/Co(8), which also has, unsurprisingly, Néel DWs. Finally, the linecut extraction technique used for the previous three samples could not be applied to Fe(3)/Co(7). In this case, owing to the higher texture density, images with varying tilt could not be reliably aligned (see § S2). Nonetheless, a visual inspection of the contrast evolution with tilt suggests that the textural helicity for Fe(3)/Co(7) is very similar to that of Fe(2)/Co(8) hosting Néel DWs.

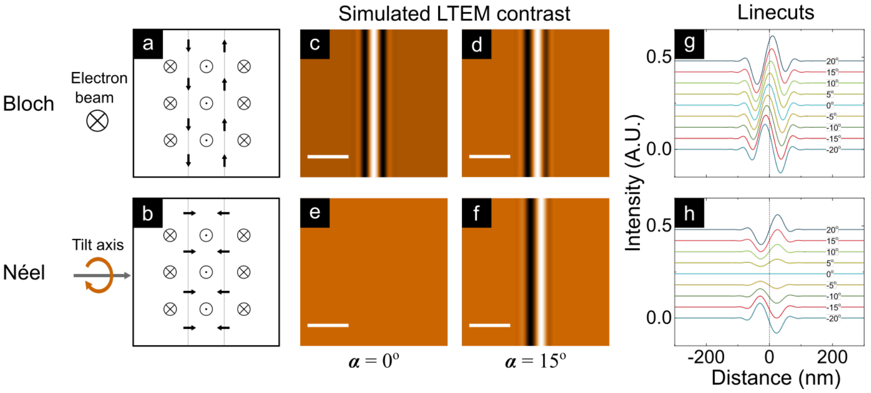

Figure S5. **Simulated LTEM contrast.** **(a-b)** Schematic of an artificially constructed magnetic stripe for MALTS simulations with Bloch and Néel DWs respectively. Vector symbols represent the direction of the electron beam and local magnetization. **(c-f)** MALTS simulated LTEM contrast at $\alpha = 0°$ and 15° (scalebar: 200 nm). **(g-h)** Linecuts across the simulated LTEM images with α varying over ± 20°.

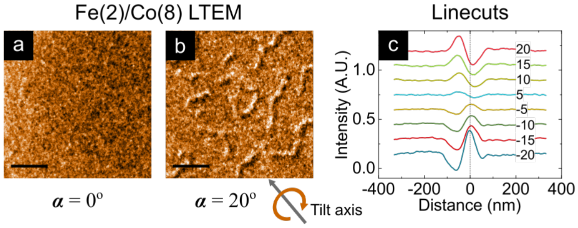

Figure S6. **LTEM analysis of DW helicity for Fe(2)/Co(8).** **(a-b)** LTEM images of Fe(2)/Co(8) sample at $\alpha = 0°$ and 20°, at a field of 100 mT and at −2 mm defocus (scalebar: 0.5 μm). **(c)** Averaged cross-sectional linecuts determined over the experimental field-of-view with tilt angle varying over ±20° (c.f. manuscript Fig. 1d-e).

Transport-of-Intensity Equation (TIE). The transport-of-intensity equation (TIE) has previously been used to reconstruct the magnetization of Bloch and Néel textures with varying degrees of success[7]. Correspondingly in Figure S7, we show the IP magnetic induction for ^{S}Co(10) and Fe(0)/Co(10), each reconstructed using two LTEM images at defoci of ±2 mm using a TIE-based commercial software, QPt^{TM} for Digital Micrograph from HREM Research, Japan (c.f. manuscript Fig. 1). For ^{S}Co(10), we can clearly see the evidence for Bloch DWs in Figure S7c. In contrast, for Fe(0)/Co(10), due to the finite tilt angle, a part of the out-of-plane magnetization of the textures are projected in-plane and is visible in Figure S7d. Crucially, no evidence of Bloch DWs is detectable for Fe(0)/Co(10).

Domain compressibility investigated with LTEM. In manuscript Fig. 2, we present MTXM results as direct experimental evidence when investigating domain compressibility as a function of κ. The same trend could be observed indirectly with LTEM. To this end, LTEM imaging was performed along a hysteresis loop, as shown in Figure S8a-d. It is visually apparent that at low fields of −20 mT, the average domain width is much greater for Fe(0)/Co(10) (Figure S8a) than that of Fe(2)/Co(8) (Figure S8c). However, at higher fields, their domain widths are comparable (Figure S8b,d). This visual observation could be placed on quantitative standing if we employ the previously established algorithmic linecut analysis, but this time as a function of the magnetic field, as shown in Figure S8e,f (c.f. MTXM in manuscript Fig. 2i). As the linecuts are centered on the intensity peaks, the domain width is simply the separation between the intensity peak (bright) and trough (dark) regions. For Fe(0)/Co(10) at low fields, the trough is separated ∼200 nm from the peak and is heavily smeared. This is because DWs are well separated (i.e. domains are wide) with weak spatial correlation. With increasing field, the trough moves towards the peak (i.e. domain width shrinks) and becomes more correlated. This is consistent with the highly compressible behavior seen in MTXM data. In contrast, for Fe(2)/Co(8), the peaks and troughs are always adjacent and have strong spatial correlation at all fields. This directly supports our claim that the domains are incompressible for

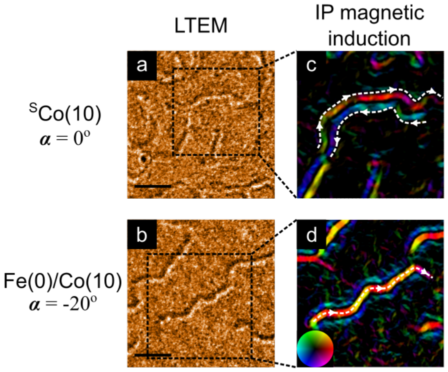

Figure S7. **Transport-of-intensity equation (TIE) analysis of LTEM images. (a,b)** LTEM images of ^{S}Co(10) and Fe(0)/Co(10) at $\alpha = 0°$ and $-20°$ respectively (scalebar: 0.5 μm). **(c,d)** Reconstructed IP magnetic induction of ^{S}Co(10) and Fe(0)/Co(10) using LTEM images acquired at defoci of ± 2 mm. The magnetization direction is represented by color. Inset to (d) shows color wheel, and white dotted lines with arrows are guides for the eye.

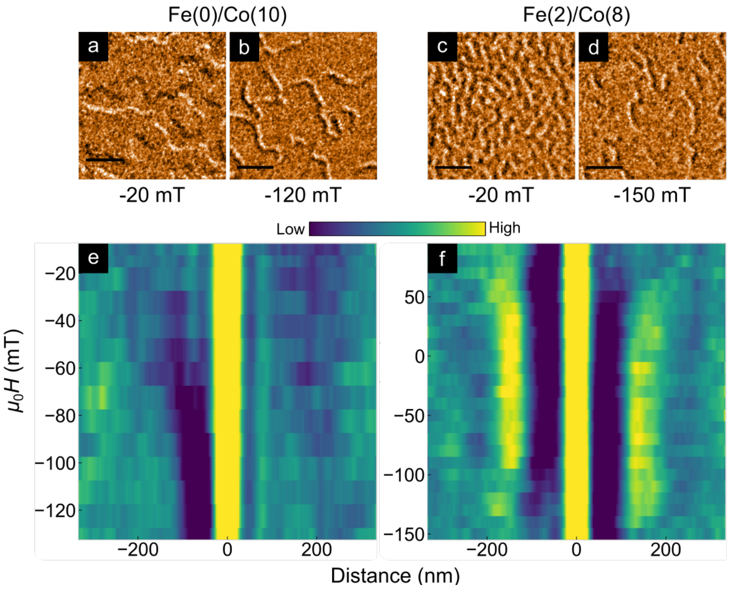

Figure S8. **Domain compressibility investigated by LTEM. (a-d)** Representative LTEM images of Fe(0)/Co(10) and Fe(2)/Co(8) at low and high magnetic fields (scalebar: 0.5 μm). **(e,f)** Stacked color plots of field-of-view averaged cross-sectional linecuts for Fe(0)/Co(10) and Fe(2)/Co(8) as a function of varying magnetic field (along y-axis). color represents LTEM intensity along the linecut.

Fe(2)/Co(8).

S4. Micromagnetic Simulations

Micromagnetic simulations were performed with MuMax3, using the magnetic parameters listed in Table S1[8].

Layer-dependent chirality. In the manuscript, we discuss the lack of evidence for hybrid chirality in Fe(0)/Co(10), Fe(2)/Co(8) and Fe(3)/Co(7) based on our LTEM data. Our micromagnetic simulations – which will be elaborated here – are consistent with our experiments in suggesting that hybrid chirality, if present at all, is very limited in these three samples.

The zero-field micromagnetic simulations were analyzed and DWs regions for each layer were isolated for analysis. Figure S9a-d shows the OP magnetization (grayscale) and IP orientation of the DW magnetization relative

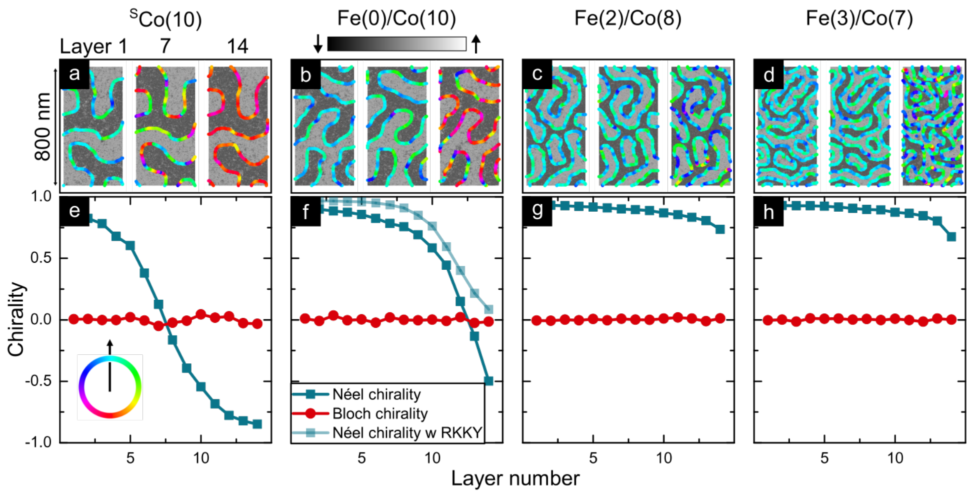

Figure S9. **Layer-dependent chirality of DWs from simulations. (a-d)** Representative cropped regions for layers 1, 7 and 14 from zero-field micromagnetic simulations of the four samples studied in this work. The grayscale colormap represents the OP magnetization, while colored ribbons show the IP magnetization orientation of the DWs relative to the DW normal vector. Inset in (e) shows the color wheel for IP orientation of DW magnetization, wherein the black arrow represents the direction of the DW normal. **(e-h)** The spatially averaged degree of Néel and Bloch chiralities as a function of layer number for the four samples. Here, the degree of Néel and Bloch chiralities are defined as $\mathbf{n}_{\mathrm{DW}} \cdot \mathbf{m}$ and $\mathbf{n}_{\mathrm{DW}} \times \mathbf{m}$ ($\mathbf{n}_{\mathrm{DW}}$ is the DW normal unit vector and $\mathbf{m}$ is the magnetization unit vector) respectively. The light teal line plot in (f) shows Bloch chirality when interlayer RKKY coupling – at 20% of direct exchange – is considered.

to the DW normal vector (colored ribbons). The dot and cross products of the magnetization unit vector with the normal unit vector of the DWs were computed, which can be identified as the degree of Néel and Bloch chiralities respectively. The averaged layer-dependent Néel and Bloch chiralities are plotted as a function of layer number for the four samples in Figure S9e-h.

Our simulations suggest that ^{S}Co(10) ($D_{\mathrm{est}} = 0$) has considerable hybrid chirality[9]. The layers near the center have Bloch helicity, while the layers near the top and bottom form Néel caps, consistent with previous works[9,10]. However, upon considering all layers as a whole, both Bloch and Néel chiralities average to zero. Therefore, we label this sample as achiral[11,12]. In comparison, the other three samples – Fe(0)/Co(10), Fe(2)/Co(8) and Fe(3)/Co(7) – with $D_{\mathrm{est}} > 0$ are strongly chiral, i.e. have fixed handedness. Even if a Bloch center is present in Fe(0)/Co(10) ($D_{\mathrm{est}} = 1.3$ mJ/m^2), it might only exist in one or two layers which is difficult to observe experimentally. Moreover, we show that Fe(0)/Co(10) is at the threshold of hybrid chirality since the Bloch center vanishes if we consider a small but finite interlayer RKKY coupling – amounting to 20% of A_{est} – as shown in Figure S9f[13]. Hence, within this work, our simulations demarcate the boundaries of hybrid chirality as $\kappa \lesssim 0.3$. Meanwhile, we do not expect a Bloch center in Fe(2)/Co(8) and Fe(3)/Co(7) ($D_{\mathrm{est}} \geq 1.8$ mJ/m^2), since their Néel chirality did not change sign across layers.

Simulation of hysteresis loops. Hysteresis loops are simulated by time-stepping in the presence of a sweeping applied magnetic field[8]. The rate of field sweep is approximately 10^6 T/s due to computational constraints, and so an entire hysteresis loop simulation is swept in about 600 ns. In order to ensure that simulated magnetization configurations may cross energy barriers at rates corresponding to conventional magnetometry experiments despite these constraints, the simulation temperature needs to be correspondingly elevated (e.g. to 850 – 900 K). A typical hysteresis loop simulation result is shown in Figure S10 for direct comparison with experiments. Notwithstanding quantitative discrepancies of $\sim$20% in saturation field and magnetization, the simulated hysteresis loops fully reproduce the key experimental features, such as the sheared shape and various kinks.

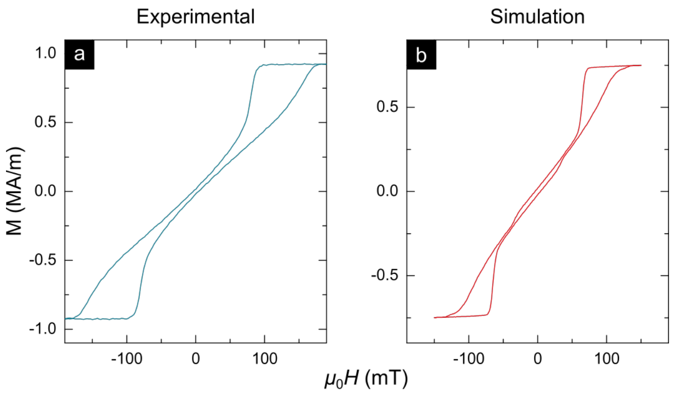

Figure S10. **Hysteresis loop simulation.** Comparison of the (a) experimental and (b) simulated OP hysteresis loop for sample Fe(2)/Co(8). Hysteresis loop simulations were performed with MuMax3 using procedures described in the text.

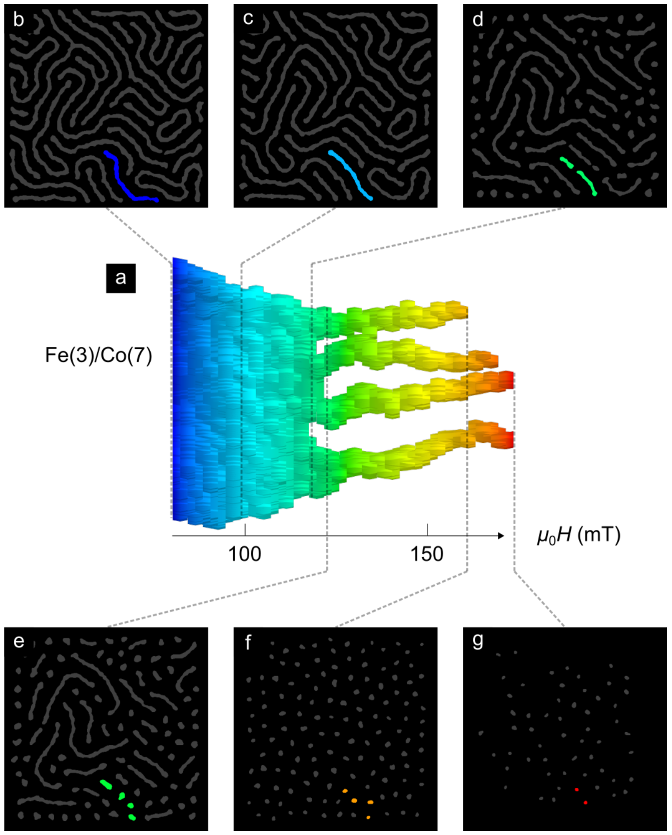

Figure S11. **Construction of a field evolution map of a typical domain.** **(a)** Field evolution map of a typical Fe(3)/Co(7) domain as shown in manuscript Figure 4b. Color denotes the magnitude of the magnetic field. **(b-g)** Simulated magnetization images (field-of-view: 2 μm) at various fields that are combined to construct (a). The chosen domain(s) is (are) highlighted in each slice.

Domain mapping. It is convenient to visualize skyrmion formation from magnetic stripes in simulations with a "field evolution map" of a typical magnetic domain. This is created by stacking the 2D footprint of a typical domain across varying fields into a 3D object, where the third axis is the magnetic field. This process is illustrated in Figure S11, where the chosen domain is in color. This 3D object is then rotated such that the field axis is horizontal, as shown in manuscript Fig. 4a,b. The mapping of the chosen domain across image slices in the magnetic field sweep is established using their spatial overlap – since the domains do not move appreciably within one field step.

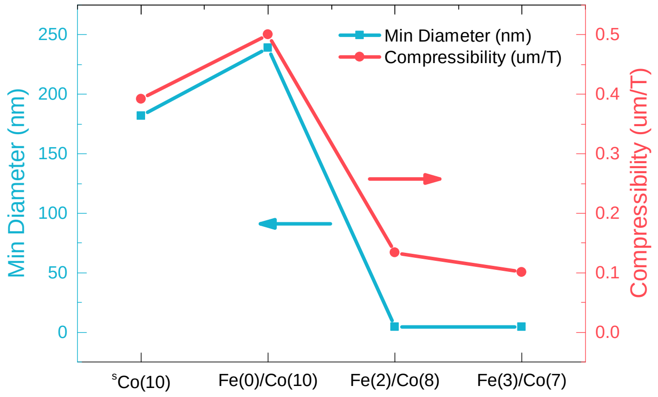

Figure S12. **Minimum skyrmion diameter predicted from isolated skyrmion model.** Theoretical results obtained by feeding in magnetic parameters listed Table S1 into model of isolated skyrmions[17]. This is compared with experimentally measured compressibility from manuscript Fig. 2k. The trends of both metrics are in close agreement – a sharp divide separates bubble skyrmions (^{S}Co(10), Fe(0)/Co(10)) from compact skyrmions (Fe(2)/Co(8), Fe(3)/Co(7)).

We also include two supplementary videos showing the evolution of the magnetic textures as a function of applied field that is used in the construction of manuscript Fig. 4(a,b).

SV1. This video shows the simulated evolution of magnetic textures of Fe(2)/Co(8) over a 2 μm field-of-view from 72 mT to 132 mT. Highlighted domains denote the representative evolution of stripes to skyrmions for Fe(2)/Co(8), and are horizontally stacked across fields to form manuscript Fig. 4(a).

SV2. This video shows the simulated evolution of magnetic textures of Fe(3)/Co(7) over a 2 μm field-of-view from 80 mT to 170 mT. Highlighted domains denote the representative evolution of stripes to skyrmions for Fe(3)/Co(7), and are horizontally stacked across fields to form manuscript Fig. 4(b).

S5. Geodesic Nudged Elastic Band (GNEB) simulations

GNEB atomistic calculations were performed with the Fidimag package[14]. A single layer of magnetic spins arranged in a two-dimensional square lattice with a cell size of 1 nm $\times$ 1 nm was used for all the GNEB simulations.

Energy barrier. GNEB is a well-established method to calculate the energy barrier for a transition between two fixed metastable magnetic configurations, under the constraint of fixed magnetic moments magnitude[15]. Since GNEB is an atomistic calculation, the energies of the configurations are calculated in accordance with the atomistic Heisenberg Hamiltonian, taking into account exchange, DMI, uniaxial anisotropy and magnetostatic interactions[16]. To estimate the energy barriers of the stripe-skyrmion fission process, the initial metastable configuration was chosen to be single stripe domain relaxed at -0.04 mT, and the final configuration to be two metastable skyrmions relaxed at the same external field. A total of 16 intermediate transition states and their energies were calculated by the GNEB algorithm, from which the energy barrier of the process can be estimated by interpolation. The resulting energy barriers of the stripe-skyrmion transition for Fe(2)/Co(8) and Fe(3)/Co(7) are shown in the main text.

S6. Compressibility

Bubble skyrmions vs compact skyrmions. Here, we compare two metrics that can be used to differentiate bubble skyrmions and compact skyrmions. In Figure S12, we show the compressibility of stripes reproduced from manuscript Fig. 2k and minimum diameter of isolated skyrmions[17] for the four samples in this work. Both met-

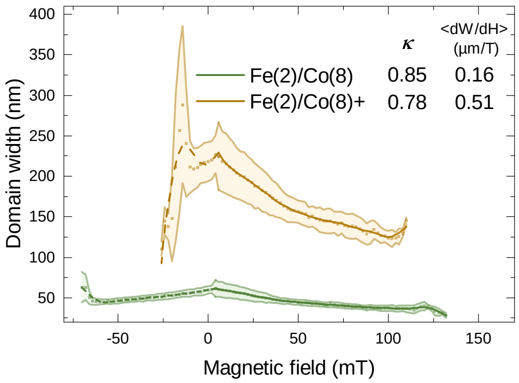

Figure S13. **Simulated domain width field evolution of Fe(2)/Co(8) and Fe(2)/Co(8)+.** Micromagnetic simulations of domain width as functions of applied magnetic field of Fe(2)/Co(8) and Fe(2)/Co(8)+, a fictitious sample with identical magnetic parameters as Fe(2)/Co(8) except an exchange stiffness that is 20% larger than Fe(2)/Co(8). Inset lists the κ and $\langle dW/dH \rangle$ values for both samples.

rics show a clear distinction between ^{S}Co(10), Fe(0)/Co(10) (bubble skrymions) and Fe(2)/Co(8), Fe(3)/Co(7) (compact skyrmions).

Correlation with K_{eff}. One might notice a correlation between domain compressibility, $\langle dW/dH \rangle$ and K_{eff}. We believe that this correlation arises from the fact that $\kappa = \pi D/4\sqrt{AK_{\text{eff}}}$, and therefore K_{eff} is a component of κ. Here, we show with micromagnetic simulations that K_{eff} cannot fully account for the domain compressibility trends. We study a fictitious sample, labeled as Fe(2)/Co(8)+, which has identical magnetic parameters as Fe(2)/Co(8) — including K_{eff} – except a direct exchange, A_{est}, that is 20% larger, which results in a smaller κ. We simulate the hysteresis loop of Fe(2)/Co(8)+, extract the domain width and compute the $\langle dW/dH \rangle$ using the same protocol as detailed in the manuscript. The comparison between Fe(2)/Co(8) and Fe(2)/Co(8)+ is shown in Figure S13. As can be seen, $\langle dW/dH \rangle$ can vary considerably even if K_{eff} is kept constant across samples.

---

* These authors contributed equally to this work.
† anjan@imre.a-star.edu.sg

[1] A. Soumyanarayanan, M. Raju, A. L. G. Oyarce, A. K. C. Tan, M.-Y. Im, A. P. Petrović, P. Ho, K. H. Khoo, M. Tran, C. K. Gan, F. Ernult, C. Panagopoulos. *Nature Materials* **2017**, *16*, 9 nmat4934.

[2] T. Böttcher, Banibrato Sinha, Burkard Hillebrands, Philipp Pirro, Xiaoye Chen, Hang Khume Tan, Robert Lazkowzki, Khoong Hong Khoo, Anjan Soumyanarayanan. *Private Communication* **2020**, *1*.

[3] S. Woo, K. Litzius, B. Krüger, M.-Y. Im, L. Caretta, K. Richter, M. Mann, A. Krone, R. M. Reeve, M. Weigand, P. Agrawal, I. Lemesh, M.-A. Mawass, P. Fischer, M. Kläui, G. S. D. Beach. *Nature Materials* **2016**, *15*, 5 501.

[4] C. Moreau-Luchaire, C. Moutafis, N. Reyren, J. Sampaio, C. a. F. Vaz, N. V. Horne, K. Bouzehouane, K. Garcia, C. Deranlot, P. Warnicke, P. Wohlhüter, J.-M. George, M. Weigand, J. Raabe, V. Cros, A. Fert. *Nature Nanotechnology* **2016**, *11*, 5 nnano.2015.313.

[5] S. v. d. Walt, J. L. Schönberger, J. Nunez-Iglesias, F. Boulogne, J. D. Warner, N. Yager, E. Gouillart, T. Yu. *PeerJ* **2014**, *2* e453.

[6] S. K. Walton, K. Zeissler, W. R. Branford, S. Felton. *arXiv:1207.2310 [cond-mat]* **2012**.

[7] S. D. Pollard, J. A. Garlow, J. Yu, Z. Wang, Y. Zhu, H. Yang. *Nature Communications* **2017**, *8* 14761.

[8] A. Vansteenkiste, J. Leliaert, M. Dvornik, M. Helsen, F. Garcia-Sanchez, B. Van Waeyenberge. *AIP Advances* **2014**, *4*, 10 107133.

[9] W. Legrand, J.-Y. Chauleau, D. Maccariello, N. Reyren, S. Collin, K. Bouzehouane, N. Jaouen, V. Cros, A. Fert. *Science Advances* **2018**, *4*, 7 eaat0415.

[10] W. Legrand, N. Ronceray, N. Reyren, D. Maccariello, V. Cros, A. Fert. *Physical Review Applied* **2018**, *10*, 6 064042.

[11] S. A. Montoya, S. Couture, J. J. Chess, J. C. T. Lee, N. Kent, D. Henze, S. K. Sinha, M.-Y. Im, S. D. Kevan, P. Fischer, B. J. McMorran, V. Lomakin, S. Roy, E. E. Fullerton. *Physical Review B* **2017**, *95*, 2 024415.

[12] X. Yu, M. Mostovoy, Y. Tokunaga, W. Zhang, K. Kimoto, Y. Matsui, Y. Kaneko, N. Nagaosa, Y. Tokura. *Proceedings of the National Academy of Sciences* **2012**, *109*, 23 8856.

[13] W. Legrand, D. Maccariello, F. Ajejas, S. Collin, A. Vecchiola, K. Bouzehouane, N. Reyren, V. Cros, A. Fert. *Nature Materials* **2019**, *19*, 1 34.

[14] M.-A. Bisotti, D. Cortés-Ortuño, R. Pepper, W. Wang, M. Beg, T. Kluyver, H. Fangohr. *Journal of Open Research Software* **2018**, *6*, 1 22.

[15] P. F. Bessarab, V. M. Uzdin, H. Jónsson. *Computer Physics Communications* **2015**, *196* 335.

[16] D. Cortés-Ortuño, W. Wang, M. Beg, R. A. Pepper, M.-A. Bisotti, R. Carey, M. Vousden, T. Kluyver, O. Hovorka, H. Fangohr. *Scientific Reports* **2017**, *7*, 1 4060.

[17] F. Büttner, I. Lemesh, G. S. D. Beach. *Scientific Reports* **2018**, *8*, 1 4464.
