# Supplementary figures and images for: Unveiling the Emergent Traits of Chiral Spin Textures in Magnetic Multilayers
Source: Adv Sci (Weinh). 2022 Jan 2;9(6):2103978. doi: 10.1002/advs.202103978 (PMC8867163; doi:10.1002/advs.202103978)

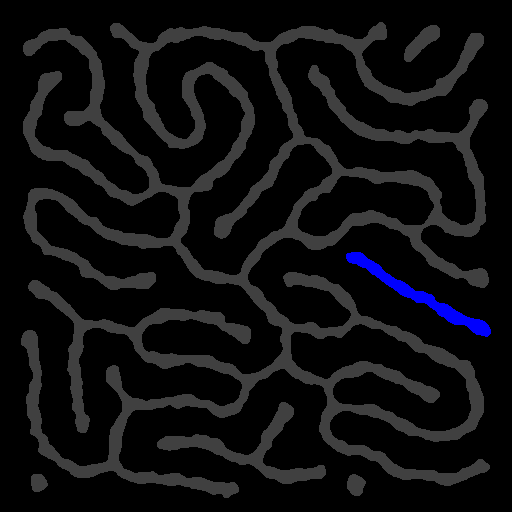

Supplement: Supplementary file 2 — Supplemental Video 1 [file ADVS-9-2103978-s001.gif]

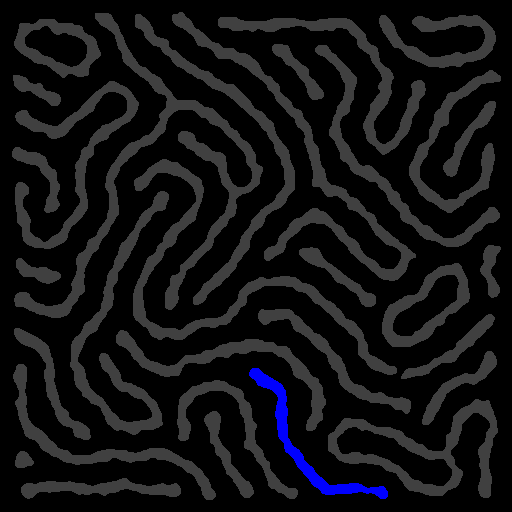

Supplement: Supplementary file 3 — Supplemental Video 2 [file ADVS-9-2103978-s003.gif]
